# Challenges in *O*-glycan engineering of plants

**DOI:** 10.3389/fpls.2012.00218

**Published:** 2012-09-20

**Authors:** Richard Strasser

**Affiliations:** Department of Applied Genetics and Cell Biology, University of Natural Resources and Life SciencesVienna, Austria

**Keywords:** glycosylation, metabolic engineering, secretory pathway, molecular farming, glycoprotein therapeutics

## Abstract

Plants are attractive alternative expression hosts for the production of recombinant proteins. Many therapeutic proteins are glycosylated with *N*- and *O*-glycosylation being the most prevalent forms of protein glycosylation. While *N*-glycans have already been modified in plants toward the formation of homogenous mammalian-type glycoforms with equal or improved biological function compared to mammalian-cell culture produced glycoproteins little attention has been paid to the modification of *O*-linked glycans. Recently, the first step of mammalian *O*-glycan biosynthesis has been accomplished in plants. However, as outlined in this short review there are important issues that have to be addressed in the future. These include: (i) elimination of potentially immunogenic or allergenic carbohydrate epitopes containing arabinosides or arabinogalactans, (ii) a detailed investigation of the interplay between engineered *N*- and *O*-glycosylation pathways to avoid competition for common metabolites like UDP-GlcNAc, and (iii) a deeper understanding of signals and mechanisms for distribution of glycan processing enzymes, which is a prerequisite for complete and homogenous glycosylation of recombinant proteins.

## INTRODUCTION

Recombinant pharmaceuticals are the fastest growing class of novel medicine with monoclonal antibodies, hormones like erythropoietin (EPO) and growth factors as the major drivers (Aggarwal, 2010). The majority of the protein-based therapeutics are glycosylated (Durocher and Butler, 2009) and the impact of different glycoforms on the function of recombinant proteins is well documented (Jefferis, 2009). Despite these facts, current mammalian cell culture-based expression systems cannot produce customized glycoforms on recombinant proteins. One reason for this inability lies in the complex mammalian glycome that consists of hundreds of glycosylation enzymes (Cummings, 2009) and produces an unwanted background glycosylation in expression hosts. Consequently, there is a growing demand for production systems that allow the control of glycosylation and generate defined homogenous glycans (Rich and Withers, 2009). Plants are an attractive alternative because they are cost-effective, highly scalable, free from human pathogens, and, importantly, can carry out post-translational modifications like glycosylation similar to mammals (Ma et al., 2003; Gomord et al., 2010; Nagels et al., 2012). *N*- and *O*-glycans are the two major carbohydrates on recombinant protein therapeutics. Both glycosylation types differ in their linkage to the protein backbone, their biosynthesis and sugar composition. Engineering of the *N*-glycosylation pathway of various plant species led to remarkable success resulting not only in the removal of immunogenic sugar moieties like β1,2-linked xylose and core α1,3-linked fucose (Koprivova et al., 2004; Strasser et al., 2004, 2008; Cox et al., 2006; Shin et al., 2011) but also in the complete reconstruction of mammalian glycosylation pathways in plants (Palacpac et al., 1999; Bakker et al., 2001; Rouwendal et al., 2009; Strasser et al., 2009; Castilho et al., 2010, 2011; Nagels et al., 2011). In contrast to that, comparatively little attempts have so far been made to modify the *O*-glycosylation machinery of plants. Here, I give a short overview of recent developments toward the production of humanized *O*-glycan structures and summarize challenges for the future.

## MUCIN-TYPE *O*-GLYCAN BIOSYNTHESIS

**FIGURE 1:**
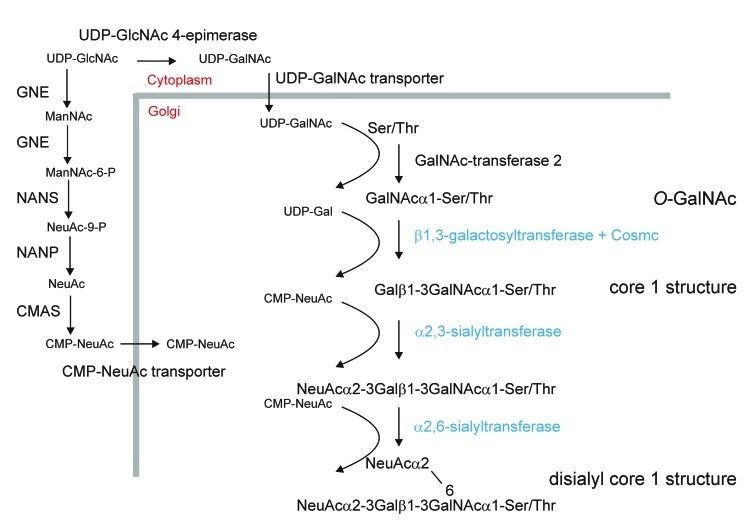
***O*-glycosylation engineering steps required to produce the disialylated core 1 structure which is, for example, the major *O*-glycan on Ser-126 of human EPO** (Tsuda et al., 1990) In addition to providing CMP-NeuAc in the Golgi and initiation of *O*-glycosylation by transfer of a GalNAc residue the biosynthesis requires the expression of a mammalian core 1 β1,3-galactosyltransferase together with its specific chaperone Cosmc as well as α2,3- and α2,6-sialyltransferases (Gill et al., 2011; Ju et al., 2011). These steps are depicted in blue and have not been engineered into plants so far. The generation of *O*-GalNAc residues has been shown (Daskalova et al., 2010; Yang et al., 2012) and the successful expression of the enzymes (GNE, UDP-*N*-acetylglucosamine 2-epimerase/*N*-acetylmannosamine kinase; NANS, *N*-acetylneuraminic acid phosphate synthase; CMAS, CMP-*N*-acetylneuraminic acid synthetase; NANP, *N*-acetylneuraminate-9-phosphate phosphatase, this dephosphorylation step is catalyzed by an endogenous plant enzyme) for CMP-sialic acid synthesis has been demonstrated in *A. thaliana* and *N. benthamiana* (Castilho et al., 2008, 2010). In total, the efficient generation of disialylated core 1 structures requires the expression of at least nine non-plant proteins. According to a recent publication the ectopic expression of a UDP-GalNAc transporter is not essential for efficient *O*-glycosylation initiation in *N. benthamiana *(Yang et al., 2012). The requirement of the specific chaperone Cosmc for core 1 β1,3-galactosyltransferase folding and activity needs to be tested in plants.

*O*-glycosylation is a common post-translational modification of serine (Ser)/threonine (Thr) residues of secreted and membrane-bound mammalian proteins. *O*-glycosylation is fundamentally different from *N*-glycosylation as a typical consensus amino acid sequence has not been clearly identified yet (Bennett et al., 2012) and *O*-glycan biosynthesis occurs in a stepwise fashion involving the sequential transfer of single sugar residues by distinct glycosyltransferases. The initiation of mucin-type *O*-glycan formation, which is the most common *O*-linked glycan in humans, encompasses the transfer of an *N*-acetylgalactosamine (GalNAc) residue from the nucleotide sugar UDP-GalNAc to hydroxyl side chains of Ser/Thr. This specific reaction is catalyzed by a family of more than 20 different polypeptide GalNAc-transferases (**Figure 1**; Ju et al., 2011; Bennett et al., 2012). Initiation of mucin-type *O*-glycosylation in the Golgi suggests that the *O*-linked structures are generated entirely post-translationally, after folding and subunit assembly has been completed in the endoplasmic reticulum. Due to the possibility of various chain elongation and branching steps there is considerably heterogeneity of mucin-type *O*-glycans. Typically these carbohydrates can be further elongated by incorporation of galactose, fucose, *N*-acetylglucosamine (GlcNAc) and sialic acid residues in different linkages (Tarp and Clausen, 2008). Single *O*-linked glycans are for example found on EPO or on interleukin-2, while other human proteins like mucin-1 (MUC1) or immunoglobulin A (IgA) contain clusters of *O*-glycans. Since specific *O*-glycans are more abundant on tumors it has been proposed that peptides containing defined *O*-glycan structures can be used as anti-cancer vaccines to elicit an immune reaction against epitopes present on cancer cells (Springer, 1984; Ju et al., 2011).

Plants lack the machinery to produce mammalian-type *O*-glycosylation (Daskalova et al., 2010; Yang et al., 2012) and recombinant EPO produced in *Nicotiana benthamiana* does not contain any *O*-linked GalNAc residues (Castilho et al., 2011). To initiate mucin-type *O*-glycan formation the corresponding mammalian GalNAc-transferase that transfers a single GalNAc residue to Ser/Thr residues has to be expressed in plants. Apart from the GalNAc-transferase and the acceptor substrate *O*-GalNAc formation depends on the presence of the nucleotide sugar in the same subcellular compartment. The glycoengineering of the *O*-glycosylation initiation step has been reported recently by two groups. Daskalova et al. (2010) could show by lectin blotting that the transfer of single GalNAc residues to a transiently expressed *O*-glycosylation substrate containing human MUC1 tandem repeats requires the expression of three non-plant proteins in *N. benthamiana*. To generate sufficient amounts of the UDP-GalNAc donor substrate and transport it into the Golgi apparatus, a microbial UDP-GlcNAc 4-epimerase and a UDP-GlcNAc/UDP-GalNAc transporter from nematodes were stably expressed together with human GalNAc-transferase 2 (**Figure 1**). A similar approach was used by Yang et al. (2012) to engineer *O*-GalNAc residues into three different reporter proteins transiently expressed in *N. benthamiana*. Structural analysis by mass spectrometry confirmed the presence of GalNAc residues in a MUC1 tandem repeat containing peptide (Yang et al., 2012). Transient expression of two different human GalNAc-transferases with partly overlapping substrate specificities resulted in high-density *O*-GalNAc formation on multiple sites of the recombinant MUC1 peptide. Interestingly, *O*-glycans were also detected without ectopic expression of a UDP-GalNAc transporter indicating that the expression of two non-plant proteins is sufficient to initiate mucin-type *O*-glycosylation in plants. A possible explanation for the discrepancy between the two studies comes from differences in the described expression levels. Both groups used transient expression of their *O*-glycosylation substrates in *N. benthamiana*, but in the first study the expression of the MUC1 reporter construct was driven by the viral-based magnICON vector system and resulted in higher expression levels (Daskalova et al., 2010; Yang et al., 2012). It is therefore possible that at lower expression an endogenous plant transporter could provide sufficient amounts of UDP-GalNAc while under high productivity conditions the nucleotide sugar pool in the Golgi is rate limiting.

Apart from *O*-GalNAc formation no further elongation or branching reactions have been reported so far in plants. One important elongation is the transfer of a galactose residue in β1,3-linkage to *O*-GalNAc to generate a core 1 *O*-glycan structure (Galβ1-3GalNAc-Ser/Thr; **Figure 1**). This reaction is catalyzed in the Golgi by core 1 β1,3-galactosyltransferase. For efficient folding human core 1 β1,3-galactosyltransferase is dependent on the assistance of a specific molecular chaperone termed Cosmc (Ju and Cummings, 2002). An ortholog of Cosmc is not present in plants and consequently it is expected that the efficient formation of core 1 *O*-glycans on plant-produced recombinant proteins requires the expression of both mammalian proteins. Alternatively, the expression of an invertebrate core 1 β1,3-galactosyltransferase that is functional in the absence of a specific chaperone and has been successfully used in an *O*-glycan engineering approach in yeast (Amano et al., 2008) could be exploited for this specific elongation step. The core 1 structure is the major *O*-glycan present on IgA1 molecules and hypogalactosylated IgA1 variants result in the elicitation of autoantibodies against galactose-deficient IgA1 proteins (Wada et al., 2010). This finding highlights the need for expression systems with controlled *O*-glycan formation on recombinant proteins to avoid adverse immunologic reactions.

Another widespread modification of mammalian *O*-glycans is the attachment of sialic acid in different linkages. Typical disialylated structures are found on glycoprotein hormones like EPO and small amounts are present in the hinge region of the IgA1 heavy chain (Mattu et al., 1998). The efficient generation of sialylated *O*-glycans involves the expression of the machinery for CMP-*N*-acetylneuraminic acid (CMP-NeuAc) biosynthesis and transport of this nucleotide sugar from the cytoplasm to the Golgi lumen and distinct sialyltransferases catalyzing the transfer of sialic acid residues from CMP-NeuAc to *O*-linked glycans (**Figure 1**). While the mammalian sialic acid biosynthesis pathway has been successfully constructed in *Arabidopsis thaliana* and *N. benthamiana* (Castilho et al., 2008, 2010), the *O*-glycan specific mammalian sialyltransferases required for the generation of sialylated *O*-glycans have so far not been expressed in plants.

## ENDOGENOUS PLANT *O*-GLYCOSYLATION

Mammalian *O*-GalNAc attachment sites are exposed on the protein surface and frequently contain nearby proline residues. Expression of recombinant glycoproteins with putative *O*-glycosylation sites in plants revealed the presence of plant-specific glycosylation. In particular, plants convert the proline residues to hydroxyproline and attach arabinose residues to recombinant proteins (Karnoup et al., 2005; Pinkhasov et al., 2011). These non-human glycans can seriously hamper the broad use of plant-made therapeutics since single arabinosyl residues linked to hydroxyprolines can constitute an IgE binding epitope and thus could play a role in allergic reactions (Leonard et al., 2005). It is currently unclear whether the plant-specific glycosylation of hydroxyprolines and mammalian glycosyltransferases compete for adjacent acceptor sites (Daskalova et al., 2010; Pinkhasov et al., 2011; Yang et al., 2012). Hydroxyproline residues have been found on MUC1 peptides expressed in *N. benthamiana* in the presence and absence of the *O*-glycosylation machinery necessary for the transfer of GalNAc residues (Yang et al., 2012). The effect of mammalian-type *O*-glycosylation on arabinosylation is unclear since no arabinose residues were detected on the expressed recombinant protein. Moreover, the prolyl 4-hydroxylases (P4Hs) that are responsible for the conversion of proline to hydroxyproline in tobacco and related species are not well described (Yuasa et al., 2005). However, several members of the *A. thaliana* P4H family have been characterized recently (Velasquez et al., 2011), which makes it now practicable to screen for P4H candidates that hydroxylate specific proline residues on recombinant glycoproteins and eliminate them from expression hosts similar to the successful removal of immunogenic sugar residues from *N*-glycans (Strasser et al., 2004, 2008). Such a strategy very likely requires the elimination of several P4Hs with overlapping substrate specificities and due to possible effects on cell wall assembly (Velasquez et al., 2011) it might be necessary to perform tissue-specific knockouts or knockdowns to avoid problems with biomass formation and overall growth of the plants. Apart from preventing P4Hs expression there have also been attempts to block the formation of hydroxyproline residues using inhibitors. The ferrous chelator 2,2′-dipyridyl, which is a potent inhibitor of P4Hs was recently used in tobacco seedlings to reduce arabinogalactosylation of endogenous proteins (Moriguchi et al., 2011). Such a chemical inhibition strategy could be quite useful for transient expression but is less suitable for the stable expression of *O*-glycosylated recombinant proteins.

## *N*- AND *O*-GLYCAN ENGINEERING

Many valuable recombinant glycoproteins contain both *N*- and *O*-glycans. For example, IgA1 molecules carry in the hinge region a number of potential *O*-glycosylation sites, while other domains of the heavy chain are subjected to *N*-glycosylation (Ju et al., 2011). Human EPO contains three *N*-linked glycans along with a single *O*-linked glycan at Ser-126 and also other human plasma proteins that might be future candidates for plant-based production systems display several *N*- and *O*-glycans that contribute to their function. Consequently, we are facing the challenge to simultaneously modify both pathways in plants to achieve complete human-like glycosylation. Engineering of *N*-glycosylation on recombinant proteins involves the elimination of β1,2-xylose and core α1,3-fucose, the incorporation of β1,4-galactose as well as terminal sialic acid residues and branching of *N*-glycans.

For both *N*- and *O*-glycosylation engineering, UDP-GlcNAc is the common starting metabolite for the generation of nucleotide sugars (UDP-GalNAc, CMP-NeuAc) required as donor substrates for the different glycosyltransferases like GalNAc-transferases and sialyltransferases (**Figure 1**). Moreover, UDP-GlcNAc is also used as nucleotide sugar by endogenous *N*-acetylglucosami- nyltransferases and mammalian *N*-acetylglucosaminyltransferases that initiate branching of *N*-glycans when expressed in plants (Strasser et al., 1999; Castilho et al., 2011; Nagels et al., 2011). In mammals it is well documented that nucleotide sugar biosynthesis is tightly regulated and subjected to feedback regulation. So far there is no evidence that the engineered changes in the flux of nucleotide sugars are deleterious for plants. Castilho et al. (2008) have shown that stable transformed *A. thaliana* plants tolerate the generation of high amounts of CMP-sialic acid and its precursor without any drastic effect on plant growth and development. Importantly, neither in *A. thaliana* nor in *N. benthamiana* does the expression of the CMP-sialic acid pathway proteins result in alterations of GlcNAc levels on complex *N*-glycans from recombinant proteins. Consistent with that, no phenotypic changes have been reported in any plant that has been subjected to *N*- or *O*-glycan engineering (Castilho et al., 2008, 2010; Daskalova et al., 2010; Nagels et al., 2011; Yang et al., 2012) indicating that plants can efficiently maintain intracellular pools of different nucleotide sugars for the synthesis of various glycoconjugates. Although GalNAc has not been detected in any plant glycan so far, the presence of the corresponding nucleotide sugar UDP-GalNAc has been shown in *A. thaliana* suspension cells and in fenugreek endosperm tissue (Alonso et al., 2010). In both species the UDP-GlcNAc levels are three to four times higher than the UDP-GalNAc levels. However, the corresponding concentrations in tissues that are used for expression of recombinant proteins like *N. benthamiana* leaves and the steady-state concentration of UDP-GalNAc or other nucleotide sugars in the Golgi are completely unknown. In view of the fact that the recent studies on *O*-glycan engineering did not analyze *N*-glycans of recombinant or endogenous proteins its effect on the *N*-glycosylation capacity of plants remains to be shown. Clearly, further research is needed to better understand the nucleotide sugar interconversion reactions and mechanisms that control their biosynthesis and subcellular distribution, which will be necessary to achieve complete and highly homogenous glycosylation in plant expression systems that are subjected to intense *N*- and *O*-glycoengineering.

## GOLGI ORGANIZATION OF GLYCOSYLATION ENZYMES

All secreted recombinant glycoproteins pass through the Golgi where they acquire their final glycosylation by Golgi-resident enzymes that are distributed in sequential Golgi cisternae. The targeting signals and mechanisms that regulate the organization of glycosylation enzymes in the early secretory pathway of plants are still poorly understood (Schoberer and Strasser, 2011). A common feature of the Golgi-located glycosylation enzymes is their type II membrane protein topology. For several plant glycosyltransferases and glycosidases it has been demonstrated that the Golgi-targeting information is present in their *N*-terminal cytoplasmic, transmembrane, and stem (CTS) region without any contribution from the large luminal catalytic domain (Essl et al., 1999; Saint-Jore-Dupas et al., 2006; Schoberer and Strasser, 2011). Studies on the generation of β1,4-galactosylated *N*-glycans revealed that sub-Golgi targeting of the human β1,4-galactosyltransferase is slightly different between plant and mammalian cells leading to aberrant glycosylation (Palacpac et al., 1999; Bakker et al., 2001; Strasser et al., 2009). Similar results were observed for mammalian *N*-acetylglucosaminyltransferases (Castilho et al., 2011; Nagels et al., 2011). Replacement of their *N*-terminal domains with targeting regions from Golgi-resident plant glycosylation enzymes resulted in a more homogenous glycosylation pattern on recombinant proteins. It is apparent from these results that further approaches to elongate and branch *O*-glycans need to address the precise localization of the glycosylation enzymes within the Golgi. Given the sequential nature of *O*-glycan biosynthesis it appears essential to organize glycosyltransferases into some kind of assembly line along the Golgi apparatus. Such an ordered distribution of glycosylation enzymes across Golgi stacks has been experimentally shown for endogenous plant enzymes involved in *N*-glycan processing (Schoberer et al., 2010). However, known targeting regions might not be sufficient to fulfill this goal as there is, for example, currently only one plant-derived CTS region for *trans*-Golgi localization of glycosyltransferases available (Strasser et al., 2007; Schoberer and Strasser, 2011). It is therefore fundamental to increase our understanding of the glycosylation enzyme organization and cargo transport within the Golgi. Especially, more efforts are required to decipher sub-Golgi targeting motifs present in the CTS region of glycosylation enzymes and identify key molecular players that control their subcellular localization.

## CONCLUSION

Developments in the last 10 years have demonstrated that plants are well suited for the production of recombinant proteins with homogenous human-like *N*-glycosylation. The recent progress in initiation of mucin-type *O*-glycan formation indicates that plants are also amenable to engineering of defined mammalian-type *O*-glycosylation. However, as summarized here there are still limitations associated mainly with endogenous plant-specific glycosylation, cross-talk between engineered pathways and our restricted tools for compartmentalization of Golgi-located glycan processing enzymes that have to be overcome in the future. With the rapid progress in this field, the combination of *N*- and *O*-glycan modification steps is only a matter of time. Furthermore, current efforts in characterization of hydroxyproline formation and Golgi-targeting sequences will help to optimize the current engineering approaches and ultimately will result in the generation of efficient plant-based production platforms for glycoprotein therapeutics.

## Conflict of Interest Statement

The author declares that the research was conducted in the absence of any commercial or financial relationships that could be construed as a potential conflict of interest.

## Abbreviations

EPO: erythropoietin
GalNAc-transferase: UDP-GalNAc:polypeptide *N*-acetylgalactosaminyltransferase
Hyp: hydroxyproline
P4H: prolyl 4-hydroxylase
